# P-1825. Harm Reduction In US Veterans Who Inject Drugs: A Nationwide Cohort Study

**DOI:** 10.1093/ofid/ofaf695.1994

**Published:** 2026-01-11

**Authors:** Audun J Lier, Elliott Lowy, Lauren Beste

**Affiliations:** Northport VA Medical Center, Northport, New York; VA Puget Sound, Seattle, Washington; VA Puget Sound Health Care System, Seattle, Washington

## Abstract

**Background:**

Persons who inject drugs (PWID) are at increased risk for acquiring HIV, hepatitis C virus (HCV), and severe injection related infections (SIRI). There is sparse data about Veterans with a history of injection drug use (IDU) who access care through the Veterans Health Administration (VHA). This study aimed to identify a cohort of Veterans with evidence of IDU in order to assess clinical outcomes and harm reduction receipt.

**Table 1.**
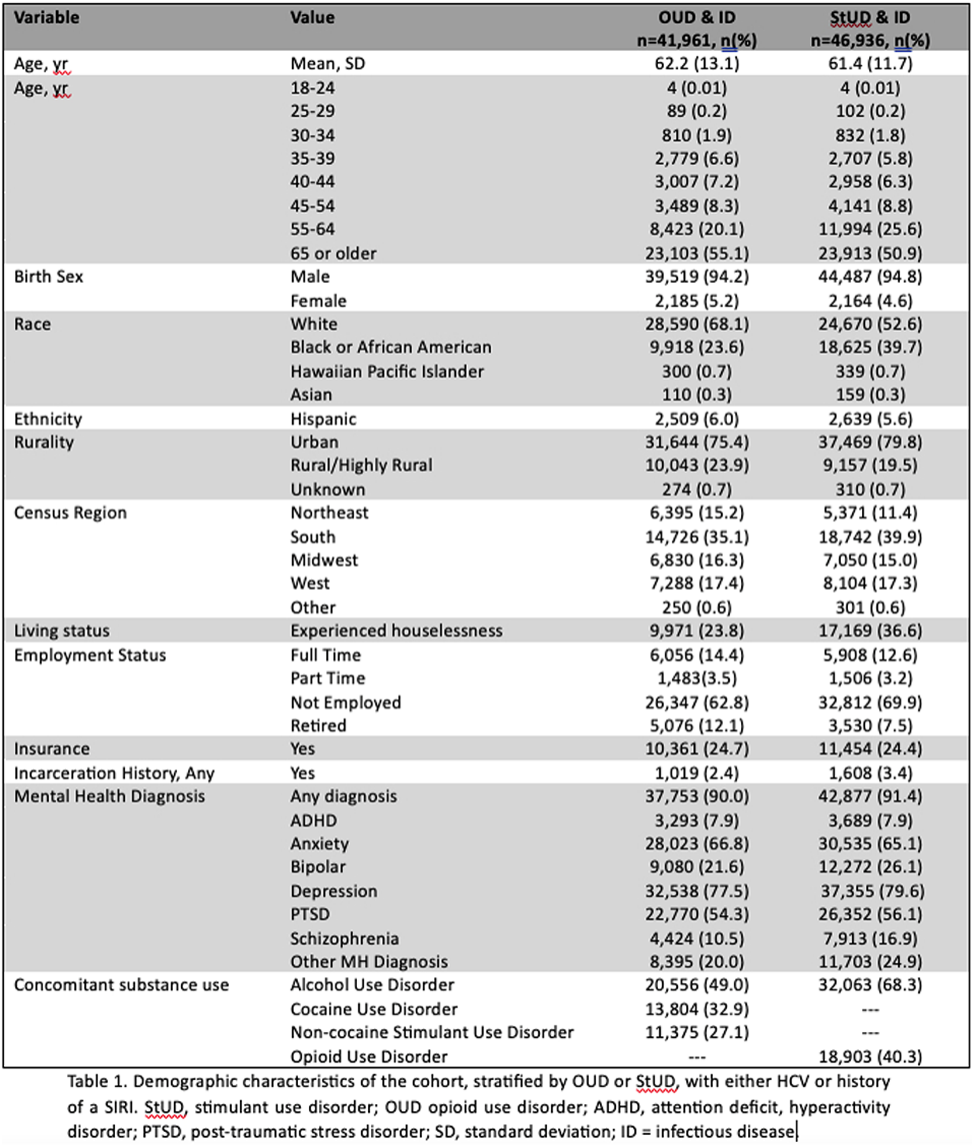
Demographic characteristics of the cohort, stratified by OUD or StUD, with either HCV exposure or a SIRI.

**Table 2.**
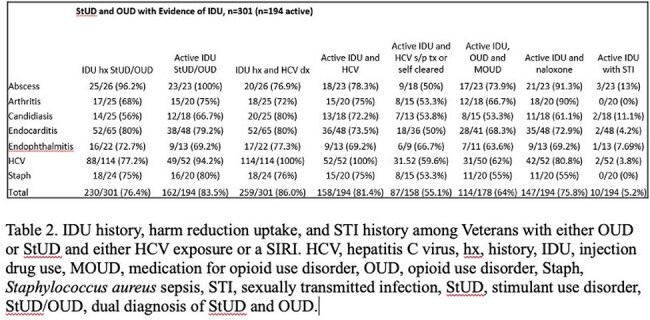
IDU history, harm reduction uptake, and STI history among Veterans with either OUD or StUD and either HCV exposure or a SIRI.

**Methods:**

A retrospective study was conducted of data obtained through the VHA Corporate Data Warehouse. Veterans who visited the VHA between 2016-2022 with an international classification of diseases, tenth edition (ICD-10) diagnosis of opioid use disorder (OUD) or stimulant use disorder (StUD), and a diagnosis of acute or chronic HCV or a SIRI were included. Cohort fidelity was assessed through a random sample of 560 Veterans. Active or remote IDU history was confirmed via text within the electronic health record. Demographics, rurality, census region, living and employment status, insurance, incarceration history, mental health and substance use histories were obtained. Harm reduction receipt was assessed.

**Table 3.**
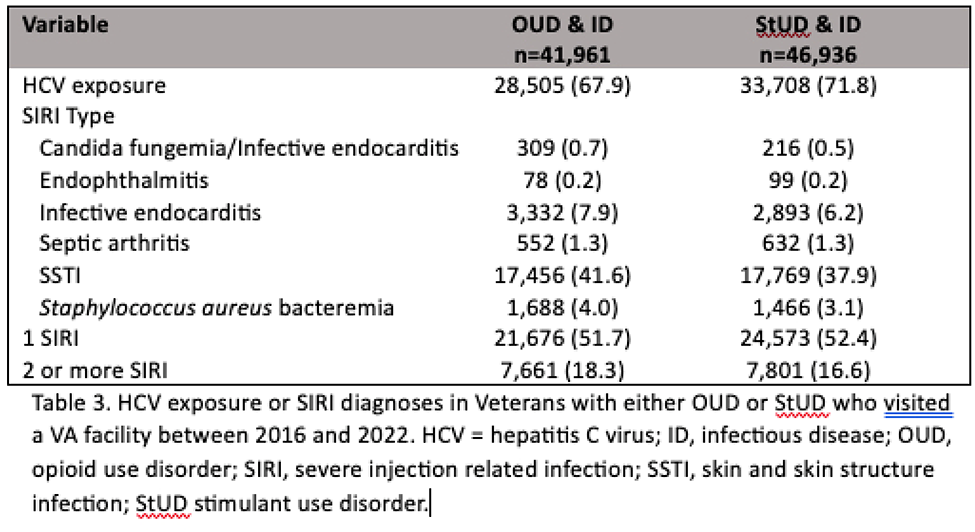
HCV exposure or SIRI diagnoses in Veterans with either OUD or StUD who visited a VA facility between 2016 and 2022.

**Results:**

There were 41,961 Veterans with OUD and HCV or a SIRI, and 46,936 Veterans with StUD and HCV or a SIRI. The OUD and StUD cohorts had a mean age of 62.2 and 61.4 years and was predominately male, White, and non-Hispanic. The highest census region was in the southern United States and in urban settings. HCV exposure was found in 28,505 (67.9%) Veterans with OUD and 33,707 (71.8%) Veterans with StUD. In the randomly selected cohort, 301 (53.8%) Veterans had a IDU history and 194 (34.6%) had active IDU. HCV exposure was found in 259 (86%) Veterans with a IDU history and 158 (81.4%) with active IDU. In Veterans with active IDU, 87 (55.1%) were treated for HCV or self-cleared, 114 (64%) were prescribed a medication for opioid use disorder (MOUD), and 147 (75.8%) were prescribed naloxone. Ten (5.2%) had a prior STI.

**Conclusion:**

A significant proportion of Veterans with OUD or StUD have a history of IDU. Veterans with active IDU may benefit from expanded efforts to increase MOUD and naloxone uptake, STI testing, treatment and PrEP for HIV in conjunction with syringe services programs. HCV exposure has a strong concordance with a history of IDU.

**Disclosures:**

All Authors: No reported disclosures

